# Enhanced Solubility and Anticancer Potential of Mansonone G By β-Cyclodextrin-Based Host-Guest Complexation: A Computational and Experimental Study

**DOI:** 10.3390/biom9100545

**Published:** 2019-09-27

**Authors:** Panupong Mahalapbutr, Piyanuch Wonganan, Thanapon Charoenwongpaiboon, Manchumas Prousoontorn, Warinthorn Chavasiri, Thanyada Rungrotmongkol

**Affiliations:** 1 Structural and Computational Biology Research Unit, Department of Biochemistry, Faculty of Science, Chulalongkorn University, Bangkok 10330, Thailand; p.mahalapbutr@gmail.com; 2 Department of Pharmacology, Faculty of Medicine, Chulalongkorn University, Bangkok 10330, Thailand; piyanuch.w@chula.ac.th; 3 Starch and Cyclodextrin Research Unit, Department of Biochemistry, Faculty of Science, Chulalongkorn University, Bangkok 10330, Thailand; thanapon.charoenwongpaiboon@gmail.com (T.C.); manchumas.h@chula.ac.th (M.P.); 4 Center of Excellence in Natural Products Chemistry, Department of Chemistry, Faculty of Science, Chulalongkorn University, Bangkok 10330, Thailand; warinthorn.c@chula.ac.th; 5 Ph.D. Program in Bioinformatics and Computational Biology, Faculty of Science, Chulalongkorn University, Bangkok 10330, Thailand; 6 Molecular Sensory Science Center, Faculty of Science, Chulalongkorn University, Bangkok 10330, Thailand

**Keywords:** beta-cyclodextrins, inclusion complex, mansonone G, molecular dynamics simulation, lung cancer

## Abstract

Mansonone G (MG), a plant-derived compound isolated from the heartwood of *Mansonia gagei*, possesses a potent antitumor effect on several kinds of malignancy. However, its poor solubility limits the use for practical applications. Beta-cyclodextrin (βCD), a cyclic oligosaccharide composed of seven (1→4)-linked α-D-glucopyranose units, is capable of encapsulating a variety of poorly soluble compounds into its hydrophobic interior. In this work, we aimed to enhance the water solubility and the anticancer activity of MG by complexation with βCD and its derivatives (2,6-di-*O*-methyl-βCD (DMβCD) and hydroxypropyl-βCD). The 90-ns molecular dynamics simulations and MM/GBSA-based binding free energy results suggested that DMβCD was the most preferential host molecule for MG inclusion complexation. The inclusion complex formation between MG and βCD(s) was confirmed by DSC and SEM techniques. Notably, the MG/βCDs inclusion complexes exerted significantly higher cytotoxic effect (~2–7 fold) on A549 lung cancer cells than the uncomplexed MG.

## 1. Introduction

Cancer is a major public health issue and is ranked as the second leading cause of mortality worldwide following cardiovascular disease [1]. Several plant-derived compounds containing naphthoquinone (NQ) moiety such as beta-lapachone, plumbagin, and shikonin have been reported to exert a superior antiproliferative activity [2,3,4]. Mansonones, group of *ortho*-NQ-containing compounds, are the major bioactive constituents of the diverse plant genera, including *Mansonia*, *Hibiscus*, and *Thespesia* [5]. Previous studies revealed that mansonones D, E, F, and H possess the anticancer effect on several kinds of solid tumors [6,7,8]. In addition, the novel derivative of mansonone F exhibited 20-fold stronger DNA topoisomerase II inhibitory activity than the chemotherapeutic drug etoposide [9].

**Figure 1:**
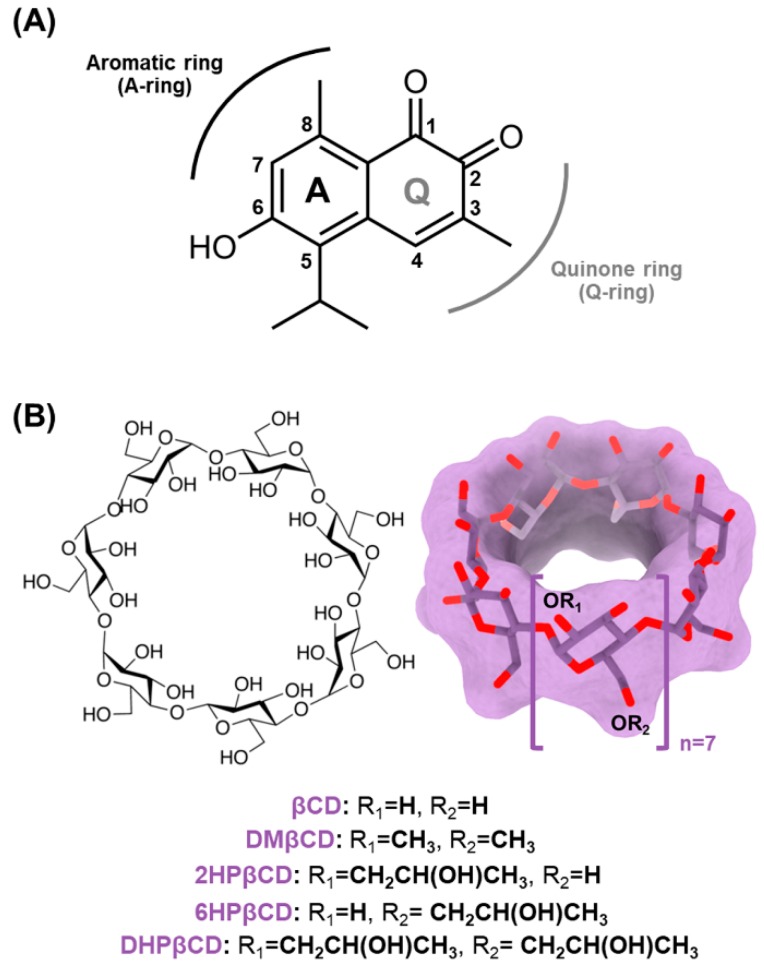
(**A**) 2D structure of MG. (**B**) 2D and 3D structures of βCD, where the functional substitutions used in this study are shown below.

Among the eight different mansonones (mansonones A–H) [10], mansonone G (MG, Figure 1A) is the major product isolated from the heartwood of *Mansonia gagei* Drumm., Sterculiaceae family found in Thailand [11]. Many lines of evidence have shown that MG exhibits a potent anticarcinogenic effect on various types of malignancies, e.g., ovarian (A278, IC_50_ of 10.2 μM), colorectal (HCT116, IC_50_ of 63.4 µM), liver (HepG2, IC_50_ of 36.3 µM and Huh-7, IC_50_ of 25.9 µM), breast (MCF-7, IC_50_ of 23.0 µM), and cervical (HeLa, IC_50_ of 18.8 µM) cancer cell lines [5,12,13], and it has been documented to significantly inhibit the activity of P-glycoprotein efflux pump [5]. Recently, the butoxy MG has shown to potentially induce cell apoptosis in human lung cancer cell lines A549 and H1975 by inhibiting STAT3 and Akt signaling pathways [14]. Even though MG could serve as a promising anticancer agent, its poor water solubility (1.7 mg/L at 30 °C) leads to a limited use for pharmaceutical and medicinal applications.

Cyclodextrin (CD), a naturally occurring cyclic oligosaccharide produced by CD glucanotransferase (CGTase)-catalyzed starch degradation [15], has a unique structure representing a hydrophobic inner cavity and a hydrophilic outer surface. Consequently, CD can potentially enhance the solubility, stability, and pharmacological properties of many lipophilic guest molecules [16,17] through the formation of an inclusion complex driven mainly by van der Waals (vdW) interactions [18]. The common types of CD are alpha (α), beta (β), and gamma (γ) CDs formed by six, seven, and eight α-D-glucopyranose units, respectively. Among the three CDs, βCD (Figure 1B) is the most commonly used in many pharmaceutical purposes due to its low price, easy synthetic access, and structural orientation suitable for inclusion complex generation [19,20]. However, the low water solubility (18.5 mg/mL at 25 °C) and nephrotoxicity of βCD limit its use for practical applications [21].

In recent years, the use of βCD derivatives (e.g., methylated (M) and 2-hydroxypropylated (HP) βCDs) for host-guest encapsulation has gained much attention in an attempt to improve the water solubility and to reduce the limitations of natural βCD. The MβCD (e.g., 2,6-di-*O*-methyl-βCD; DMβCD) and the HPβCD derivatives exhibit higher aqueous solubility and lower toxicity than the unmodified βCD [21,22,23]. Several experimental and theoretical reports (both classical molecular dynamics (MD) and density functional theory (DFT)) have demonstrated that the water solubility, chemical stability, and anticancer activity of poorly soluble molecules are significantly improved by complexation with DMβCD and HPβCD derivatives [24,25,26,27,28]. However, the information on the inclusion complexes of MG with βCDs has never been previously reported. Therefore, the present study aimed to enhance the physical and biological properties of MG using βCD and its derivatives (DMβCD and HPβCD) based on working hypothesis that the anticancer activity of MG may be positively affected by βCDs. The all-atom MD simulations and binding free energy calculations were firstly applied (*i*) to search for the suitable βCD encapsulating molecule for MG and (*ii*) to investigate the atomistic insights into host-guest complexation and its underlying interactions. Subsequently, the phase solubility study between MG and βCDs was experimentally verified, and the physical and chemical characterization techniques were used to confirm the inclusion complex formation. Finally, the resulting MG/βCDs complexes were subjected to evaluate the antitumor activity toward A549 human lung cancer cell line in comparison to the MG alone.

## 2. Materials and Methods 

### 2.1. Computational Details

The 3D structures of MG and all the investigated βCD analogs (βCD, DMβCD, 2HPβCD, 6HPβCD, and DHPβCD) were taken from previous studies [29,30,31]. It should be noted that (*i*) the hydroxyl group of MG is protonated (neutral form) based on NMR data from previous studies [11,32] and (*ii*) the commercially available HPβCD is a mixture of various degree of substitution (DS), while the models we built in this study were fully (DHPβCD; DS = 14) and partially (2HPβCD and 6HPβCD; DS = 7) substituted HPβCDs. The inclusion complexes were generated in Accelrys Discovery Studio 2.5 (Accelrys Software Inc., San Diego, CA, USA) by docking protocols using CDOCKER module. The partial charges of MG molecule were created as per the standard procedures [33,34,35]. The Glycam-06 [36] and general AMBER force fields [37] were applied on βCDs and MG, respectively. Using a truncated octahedral box, the TIP3P water molecules were used to solvate around MG/βCDs complexes with a spacing distance of 15 Å. After that, the added water molecules were minimized using 1000 steps of steepest descent and continued by 3000 steps of conjugated gradient. Lastly, the whole model was optimized as per the same methods.

Under periodic boundary condition with a time step of 2 fs, each solvated inclusion complex was heated up from 10 K to 303 K for 60 ps, and followed by all-atom MD simulations (*NPT* ensemble, temperature of 303 K and pressure of 1 atm) using AMBER16 for 90 ns in triplicate. All chemical bonds involving hydrogen were constrained using the SHAKE algorithm [38]. The particle mesh Ewald method [39] was employed to treat charge-charge interactions with a cutoff of 12 Å. The molecular mechanics/generalized Born surface area (MM/GBSA)-based binding free energy (Δ*G*_bind, MM/GBSA_) calculation [40] was used to estimate the binding affinity of all the studied inclusion complexes.

### 2.2. Experimental Part

#### 2.2.1. Chemical Reagents

MG was extracted from *Mansonia gagei* Drumm according to the previous study [11]. βCD (MW 1134.98) was obtained from Acros Organics (Morris Plains, NJ, USA), whereas the Heptakis(2,6-di-O-methyl)-β-cyclodextrin (DMβCD; MW 1331.36), (2-Hydroxypropyl)-β-cyclodextrin (HPβCD; MW 1396; average DS is 0.5–1.3 unit of 2-hydroxypropyl group per glucose unit), dimethyl sulfoxide (DMSO), and 3-(4,5-dimethylthiazol-2-yl)-2,5-diphenyltetrazolium bromide (MTT) were purchased from Sigma-Aldrich (St. Louis, MO, USA). 

#### 2.2.2. Cell lines and Culture

A549 human lung cancer cell line was obtained from Dr. Apiwat Mutirangura (Chulalongkorn University, Thailand). A549 cells were cultured in Dulbecco’s modified Eagle’s medium (DMEM; Gibco, Grand Island, NY, USA) containing 10% fetal bovine serum (FBS; Gibco), 100 U/mL penicillin, and 100 µg/mL streptomycin (Gibco) and were maintained at 37 °C in a humidified 5% CO_2_ atmosphere.

#### 2.2.3. Phase Solubility Study and Evaluation of Thermodynamic Parameters 

Phase solubility study was conducted by following the method of Higushi and Connors [41]. Briefly, an excess amount of MG was added to βCD(s) solutions (0–10 mM). The mixtures were shaken (250 rpm) at 30 °C, 37 °C, and 45 °C for 72 h. Subsequently, the suspensions were centrifuged at 12,000 rpm for 15 min. The amount of MG presenting in solution was measured by UV-Vis spectroscopy at 415 nm. The Equation (1) was used to calculate the apparent stability constant (*K_c_*), where *S*_0_ is y-intercept.

(1) $$K_{c}=\frac{Slope}{\left[S_{o}\left(1-slope\right)\right]}$$

The Van’t Hoff plot based on Equation (2) [17] was used to identify the thermodynamic properties, including enthalpy (Δ*H*) and entropy (Δ*S*) changes of inclusion complexation, whilst the experimental Gibbs free energy (Δ*G*_bind, exp_) was obtained from Equation (3).

(2) $$\ln K_{c}=-\frac{\Delta H}{RT}+\frac{\Delta S}{R}$$

Δ*G*_bind, exp_ = Δ*H − T*Δ*S* (3)

#### 2.2.4. Inclusion Complex Preparation

A 1:1 stoichiometric ratio of MG/βCD(s) inclusion complex was prepared by the freeze-drying method. Each accurately weighed compound (73.28 mg MG, 340.49 mg βCD, 399.41 mg DMβCD, and 418.80 mg HPβCD) was dissolved in deionized water (30 mL), then the mixture was magnetically stirred at room temperature for 24 h. After that, the suspension was centrifuged (12,000 rpm for 15 min) and filtered through a 0.45 µm filter. The obtained solution was then frozen overnight at −80 °C and subsequently lyophilized using LYO-LAB (Lyophilization Systems Inc., USA) for 3 days. In addition, a 1:1 molar ratio of MG and βCD(s) was physically mixed at room temperature to obtain the physical mixtures, which were used for comparison. The resulting freeze-dried powders and physical mixtures were stored in desiccator for further use. 

#### 2.2.5. Determination of Solubility

Excess quantity of pure MG and its inclusion complexes was added to 1 mL of deionized water. The mixture was continuously stirred at 30 °C for 24 h. Subsequently, the suspension was filtered through a 0.45 µm filter, and the solubility of MG was characterized by measuring the absorbance at a wavelength of 415 nm.

#### 2.2.6. Inclusion Complex Characterization

##### Differential Scanning Calorimetry (DSC)

The thermal behavior of the MG, βCD, DMβCD, MG/βCD, and MG/DMβCD was characterized using NETZSCH DSC 204F1 Phoenix. Each solid sample (~1–2 mg) was heated from 25 °C to 300 °C in aluminum pans at a rate of 10 °C /min.

##### Scanning Electron Microscope (SEM)

The surface morphology of MG, βCD, DMβCD, the freeze-dried inclusion complexes, and the physical mixtures was analyzed using a Scanning Electron Microscope (JEOL JSM-IT500HR). Samples were coated with a thin layer of gold in vacuum before viewing under 300 times magnification. Observations were performed using an accelerating voltage of 10 kV.

#### 2.2.7. Cytotoxicity of MG toward Lung Cancer Cells

Cell viability was evaluated using MTT assay. A549 cells were seeded into 96-well plates at a density of 3 × 10^3^ cells/well. After overnight incubation, cells were treated with logarithmic concentrations (1, 3, 10, 30, and 100 µM) of MG, MG/βCD, MG/DMβCD, and MG/HPβCD for 48 h. Note that the amount of treated MG in free form and in complexes is equivalent. After that, MTT solution (5 mg/mL) was added to each well and then incubated at 37 °C for another 4 h. Subsequently, the culture medium was withdrawn and 150 µL of DMSO solution was added to dissolve formazan crystals. Finally, the absorbance was measured at 570 nm using UV-Vis spectrophotometer (Thermo Scientific, Vantaa, Finland). The half-maximal inhibitory concentration (IC_50_) was calculated using GraphPad Prism 7 software.

#### 2.2.8. Statistical Analysis

The quantitative data are expressed as mean ± standard error of mean (SEM) of three independent experiments. Differences between groups were determined using one-way analysis of variance (ANOVA) followed by a Newman-Keuls post hoc test. The *p* value of ≤0.05 was considered statistically significant. 

## 3. Results and Discussion

From docking study, MG could form two possible inclusion complexes with βCDs through its aromatic ring (A-ring, form I) or quinone ring (Q-ring, form II) insertion into the hydrophobic cavity. However, after performing all-atom MD simulations for 90 ns in triplicate on both forms (altogether 30 independent simulations), only form I of MG/βCD, MG/DMβCD, and MG/2HPβCD is likely stable, whilst the dissociation of inclusion complex is detected in the form II of all systems as well as the form I of MG/6HPβCD and MG/DHPβCD. The latter finding is consistent well with previous study showing that the HP modification on βCD at C6-position (DS = 7) led to the dissociation of the encapsulated mansonones E and H from hydrophobic inner cavity [42]. Therefore, the MG/βCD, MG/DMβCD (DS = 14), and MG/2HPβCD (DS = 7) complexes in form I were further focused in this study.

### 3.1. Ligand Mobility Inside βCD’s Hydrophobic Cavity

**Figure 2:**
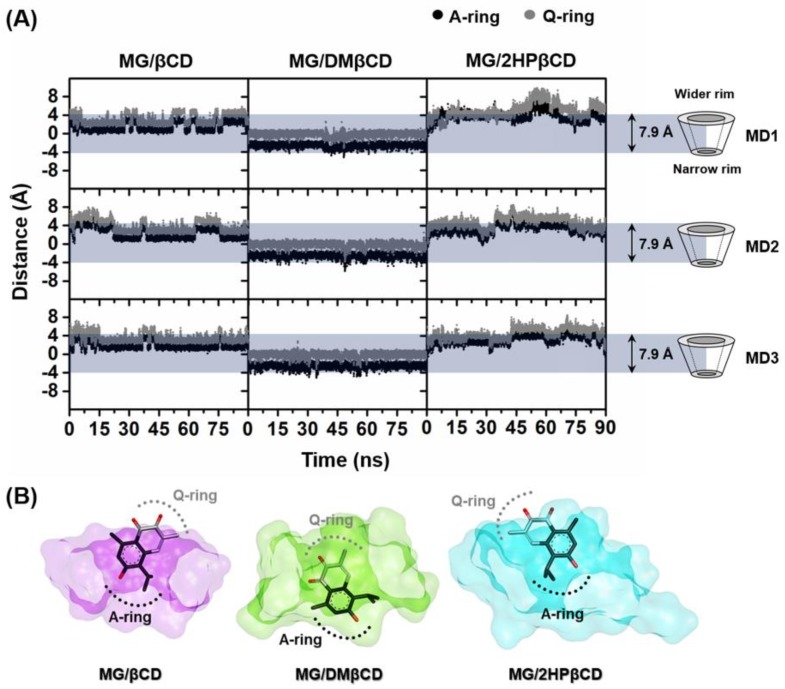
(**A**) The distance between C_m_ of MG ring (A/Q) and C_m_ of βCD of all studied inclusion complexes for three independent MD simulations (MD1-3). (**B**) The binding orientation of MG inside βCD (violet), DMβCD (green), and 2HPβCD (cyan) cavities drawn from the last MD snapshot.

The dynamics behavior of the encapsulated MG inside hydrophobic interior of βCD(s) along the simulation time was studied using the calculations of the distance between the center of mass (C_m_) of each ring of MG (A-ring (black) and Q-ring (grey)) and the C_m_ of βCD without taking into account the functional substituents. The obtained results are shown in Figure 2A, whereas the representative binding mode of MG/βCD(s) taken from the final MD snapshot is depicted in Figure 2B. The horizontal grey box ranging from −3.95Å to 3.95Å (~7.9Å) represents the positions of the primary (narrow) and secondary (wider) rims of native βCD, respectively [43]. In the case of MG/βCD and MG/2HPβCD systems, MG stably positions nearby the wider rim of βCD(s) and preferentially inserts the A-ring inside the hydrophobic inner cavity. By considering the MG/DMβCD models, MG rapidly moves toward the narrow rim of DMβCD after the heating step and subsequently positions in this orientation until the end of the simulations. 

### 3.2. βCDs Conformations Upon MG Binding

**Figure 3:**
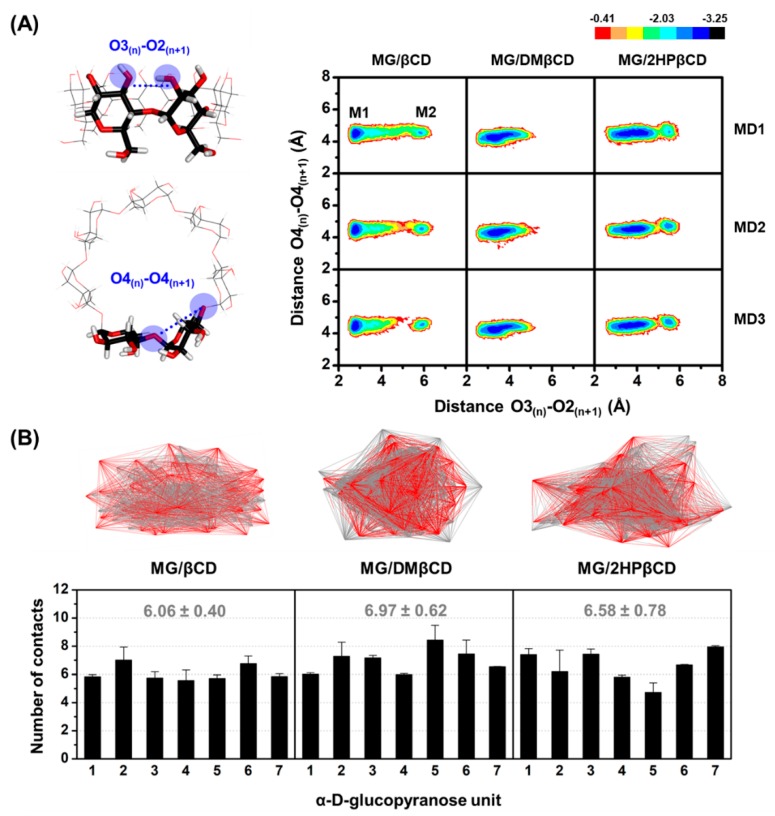
(**A**) The distance parameters (dO_3-2_ and dO_4-4_) used for PES calculations (left) and the obtained results (right). (**B**) The representative native contact PDB structures shown in line mode (top) and the number of contacts between MG and α-D-glucopyranose units of βCDs (bottom). Data are expressed as mean ± SEM of three independent MD simulations.

The βCD(s) conformational changes upon MG encapsulation were investigated by calculating (*i*) the distance of the secondary hydroxyl groups on the wider rim of βCDs (O3_(n)_–O2_(n+1)_, dO_3-2_), corresponding to a possibility of intramolecular hydrogen bond (H-bond) generation (dO_3-2_ of ≤3.5 Å), and (*ii*) the distance of glycosidic oxygen atoms (O4_(n)_–O4_(n+1)_, dO_4-4_) (Figure 3A, left). Afterward, the Equation (4) was used to transform the distributions of these two parameters into the free energy value, F(*x,y*):

F(*x,y*) = −*k*_B_*T* log[*P*(*x,y*)] (4)

where *k*_B_ is the Boltzmann constant, *T* is the temperature (303 K), and *P*(*x,y*) is the probability of dO_3-2_ (x) and dO_4-4_ (y). The potential energy surface (PES) is shown in Figure 3A, in which the M1 area is the most stable form of the βCD(s), whilst the M2 region represents the rotation of the α-D-glucopyranose units. 

As compared to the unbound form of βCDs exhibiting three local minima areas: M1 (dO_3-2_ of ~3.0–4.5 Å and dO_4-4_ of ~4.3–4.7 Å), M2 (dO_3-2_ of ~5 Å and dO_4-4_ of ~4.5–5.2 Å), and M3 (dO_3-2_ of ~5 Å and dO_4-4_ of ~6 Å) [30], the molecular encapsulation of MG toward DMβCD and 2HPβCD dramatically induces the stable conformation of βCD(s) by enhancing the formation of intramolecular H-bonds on the wider rim, as evidenced by the obviously increased population in M1 region (dO_3-2_ of ~2.5–4.5 Å and dO_4-4_ of ~4.1–4.9 Å, Figure 3A). In addition, the M3 region with both lengthened distances, which was only detected in the free form of βCDs, is completely disappeared in all MG-bound systems. These H-bond-operated conformational changes of βCD(s) upon the ligand binding correlate well with previously published research [44]. However, the distortion of glucopyranose units represented by M2 area is found in MG/βCD and MG/2HPβCD systems, but not in MG/DMβCD models, indicating that MG/DMβCD is the most stable complex, which is consistent with Δ*G*_bind, MM/GBSA_ calculations (Table 1) as well as the experimental *K_c_* and Δ*G*_bind, exp_ values (Table 2 and Table 3) as discussed later.

We further characterized the native contact points within 3 Å between MG and βCDs during the last 20-ns MD simulations. The obtained results (Figure 3B) reveal that MG/DMβCD inclusion complex displays the highest number of contacts per α-D-glucopyranose units (6.97 ± 0.62) followed by MG/2HPβCD (6.58 ± 0.78) and MG/βCD (6.06 ± 0.40) complexes, respectively, as clearly shown by the representative 3D contact structures (Figure 3B, top) demonstrating that MG/DMβCD exhibits the most compact feature. Altogether, the native-contact-driven structures consistently support the PES calculations.

### 3.3. Solvent Accessibility Toward Inclusion Complexes

**Figure 4:**
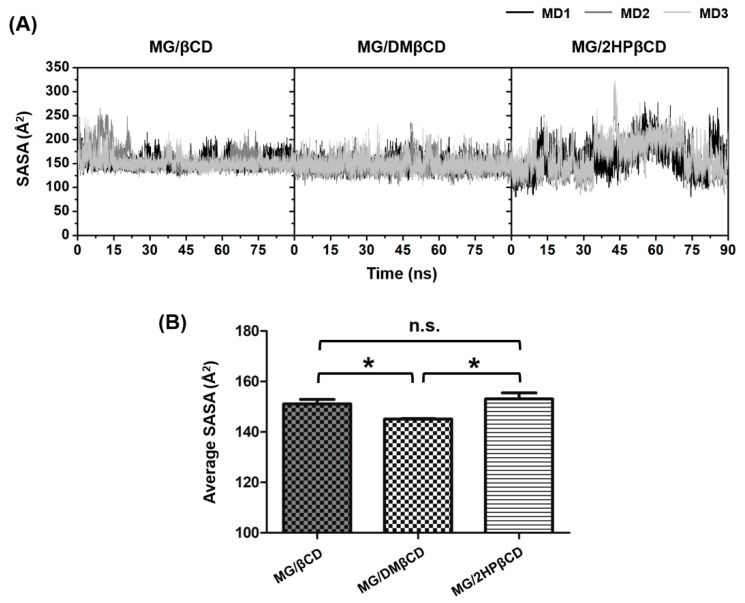
(**A**) SASA of MG/βCD, MG/DMβCD, and MG/2HPβCD for three different MD runs. (**B**) The average SASA over the last 20-ns MD simulations for each inclusion complex. Data are expressed as mean ± SEM (*n* = 3). n.s., not significantly different. * *p* ≤ 0.05.

The effect of water accessibility on MG/βCD(s) inclusion complex formation was characterized by solvent accessible surface area (SASA) calculations using MG as the atomic radii for solvent-exposed area. The entire SASA results are depicted in Figure 4A, whereas the average SASA values taken from the last 20-ns MD simulations are illustrated in Figure 4B.

Data in Figure 4A indicate that the SASAs of MG/βCD and MG/DMβCD systems are more stable than those of MG/2HPβCD complexes. In the case of MG/βCD, the SASAs fluctuate in the range of ~150–250 Å^2^, whereas the SASAs of MG/DMβCD are relatively steady at ~150 Å^2^ along the simulation times. By considering the MG/2HPβCD models, the SASA values considerably fluctuate (~200–300 Å^2^) at 40–70 ns and subsequently decrease to ~150 Å^2^ after 70 ns (except MD1). The average SASA values over the last 20 ns from three independent simulations in Figure 4B reveal that the complexation of MG with DMβCD significantly decreases the water accessibility toward the MG molecule inside hydrophobic inner cavity as compared to the MG/βCD and MG/2HPβCD (* *p* ≤ 0.05). These observations correlate well with previous study [45] demonstrating that the lowest solvent exposed inclusion complex displayed the highest binding affinity. However, the SASAs of MG/βCD and MG/2HPβCD are not significantly different, suggesting that DMβCD is the most preferred encapsulating agent for MG, which is in good correlation with MM/GBSA-based free energy calculations as discussed in the next section.

### 3.4. Binding Free Energy of Inclusion Complexes

To estimate the binding affinity of the inclusion complexes, we applied MM/GBSA [46] calculation on each MG/βCD(s) using 300 snapshots extracted from the last 20-ns MD simulations. The average Δ*G*_bind, MM/GBSA_ and its energy components, vdW (Δ*E*_vdW_) and electrostatic (Δ*E*_ele_) energies, for MG/βCDs are summarized and compared in Table 1. As expected, due to the poor solubility of MG, the host-guest complexation in gas phase is driven predominantly by vdW interactions (~ −27 to −29 kcal/mol). Similarly, the summation of Δ*G*_solv,non-polar_ + Δ*E*_vdW_ energies (~ −30 to −32 kcal/mol) is much lower than the Δ*G*_solv,polar_ + Δ*E*_ele_ terms (~8 to 10 kcal/mol), indicating the vdW forces play a pivotal role in the formation of MG/βCDs in an aqueous environment. These findings strongly correlate with those reported for other hydrophobic ligands in complex with βCDs [47,48,49].

Several studies have shown that the methyl and hydroxypropyl modifications on βCD can significantly enhance the stability of many lipophilic guest molecules [50,51,52]. In good agreement with these reports, our results demonstrate that the Δ*G*_bind, MM/GBSA_ of MG in complex with modified βCDs is significantly lower than that of MG in complex with unsubstituted βCD, which can be ranked in the order of MG/βCD (−2.34 ± 0.35 kcal/mol) > MG/2HPβCD (−3.35 ± 0.14 kcal/mol, *) > MG/DMβCD (−5.73 ± 0.04 kcal/mol, ***). Taken altogether, all structural and energetic analyses convince that DMβCD analog is the most suitable host for MG encapsulation. 

### 3.5. Phase Solubility Study and Thermodynamic Parameters

As the molecular modeling results suggest that DMβCD derivative is the most preferential host for MG, we further confirmed our findings by conducting the experimental phase solubility study and Van’t Hoff-based thermodynamic parameter evaluation.

**Figure 5:**
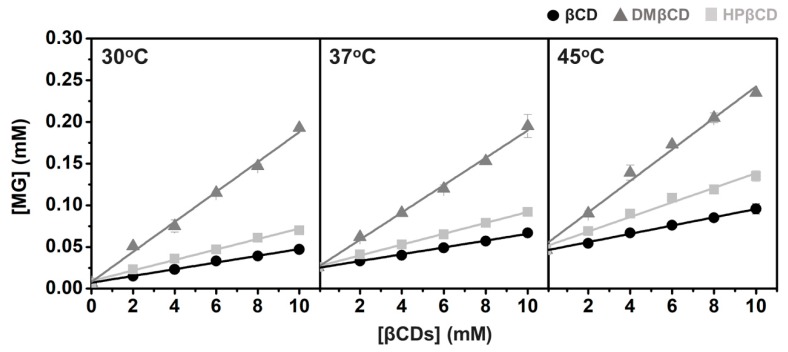
Phase solubility diagram of MG with all studied βCDs in water at 30 °C, 37 °C, and 45 °C. An excess amount of MG was added to βCD(s) solutions (0–10 mM). The mixtures were shaken (250 rpm) at 30 °C, 37 °C, and 45 °C for 72 h. Subsequently, the suspensions were centrifuged at 12,000 rpm for 15 min. The amount of MG presenting in solution was measured by UV-Vis spectroscopy at 415 nm. Data are expressed as mean ± SEM of three independent experiments.

Phase solubility diagrams of all studied inclusion complexes at temperatures 30, 37, and 45 °C are summarized in Figure 5 and Table S1, where the corresponding *K_c_* values are given in Table 2. The obtained results show that the increased βCD(s) concentrations can enhance the solubility of MG in a liner manner (ranked in the order of DMβCD >> 2HPβCD > βCD), indicating that the stoichiometric ratio between MG and βCD(s) is 1:1 (A_L_ type) [41]. By considering the stability of all studied inclusion complexes at 30 °C, the highest *K_c_* value is detected in MG/DMβCD (2245 M^−1^) followed by MG/HPβCD (684 M^−1^) and MG/βCD (562 M^−1^), respectively. Moreover, the solubility of MG is dramatically increased up to ~7 times, ~28 times, and ~10 times by complexation with βCD, DMβCD, and HPβCD, respectively (Table 4). These observations are consonant well with several reports demonstrating that βCD derivatives, especially DMβCD, could significantly enhance the stability and solubility of hydrophobic guest molecules better than natural βCD [25,53,54]. However, the increased temperature remarkably reduces the stability of all investigated complexes, in a manner similar to previous works [55,56]. 

Using Van’t Hoff plot (see Supporting Information, Figure S1), the obtained thermodynamic values, i.e., ∆*H*, ∆*S*, and ∆*G*_bind, exp_, are summarized in Table 3. The negative ∆*H* (−20.77, −23.27, and −17.39 kcal/mol for MG/βCD, MG/DMβCD, and MG/HPβCD, respectively) suggests an exothermic process. Moreover, the formation of all inclusion complexes is spontaneous, as evidenced by the negative sign of ∆*G*_bind, exp_ values at 30 °C (−3.71, −4.56, −3.85 kcal/mol for MG/βCD, MG/DMβCD, and MG/HPβCD, respectively). Notably, the trend of MM/GBSA-based free energies strongly agrees with ∆*G*_bind, exp_ values, indicating that MM/GBSA serves as a suitable method for predicting the binding affinity of MG/βCDs inclusion complexes, which is in good agreement with several studies on other host-guest systems [56,57,58,59,60].

Taken together, based on theoretical and experimental investigations, we selected the MG/DMβCD, showing the highest stability, for further structural characterizations in comparison to the MG/βCD and the free form of MG. 

### 3.6. Inclusion Complex Characterization

#### 3.6.1. Thermal Behavior of MG and Its Inclusion Complexes

**Figure 6:**
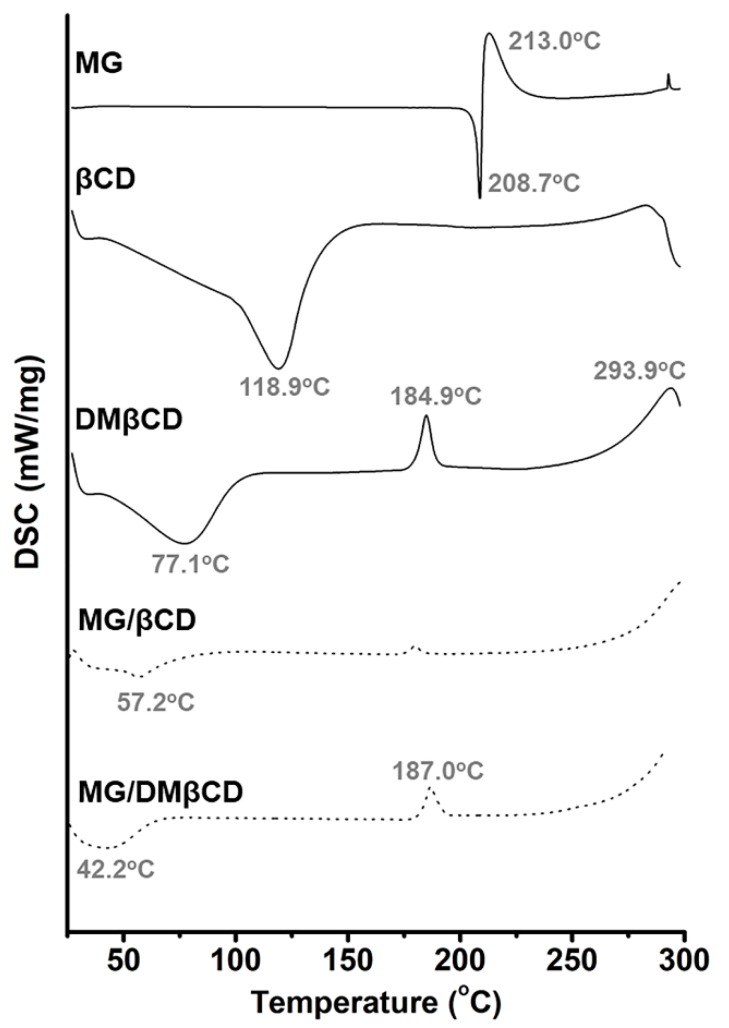
DSC thermogram of MG, βCD, DMβCD, and the freeze-dried inclusion complexes (MG/βCD and MG/DMβCD). Note that prior to perform DSC, all compounds were dissolved in deionized water and were subsequently lyophilized for 3 days.

The thermal behaviors of the starting materials (MG, βCD, and DMβCD) and the inclusion complexes (MG/βCD and MG/DMβCD) were characterized in the solid state by DSC analysis (Figure 6). The characteristic endothermic/exothermic peaks of the free compounds are as follows: MG at 208.7 °C and 213.0 °C, βCD at 118.9 °C, and DMβCD at 77.1 °C, 184.9 °C, and 293.9 °C. The broad endothermic peak of βCD detected at 118.9 °C corresponds to the release of water molecules from the hydrophobic cavity [61]. As shown in supplemental Figure S2, the distinct thermal peaks of the free MG were expressed in all the physical mixture products, indicating that the physical mixing method does not provide a real inclusion complex.

As a host-guest complexation process is occurred, the thermal features of the acquired product are dramatically changed [62]. In accordance with these facts, our data in Figure 6 reveal that the thermal peaks of the free MG were totally disappeared in all the freeze-dried inclusion complexes. In addition, in the case of MG/βCD inclusion complexation, the characteristic broad peak of βCD at 118.9 °C is shifted to 57.2 °C by the influence of the MG-induced water replacement. The similar change of dehydration peak of βCD after ligand complexation was also found by Rajendiran and coworkers [63]. Similarly, the broad endothermic peak (77.1 °C) and the exothermic peaks (184.9 °C and 293.9 °C) of DMβCD are dramatically shifted with a decreased intensity after complexing with MG, in good agreement with previous study [64]. Therefore, the freeze-drying method can successfully generate the new solid phase between MG and βCDs.

#### 3.6.2. Surface Morphological Changes upon Inclusion Complexation

**Figure 7:**
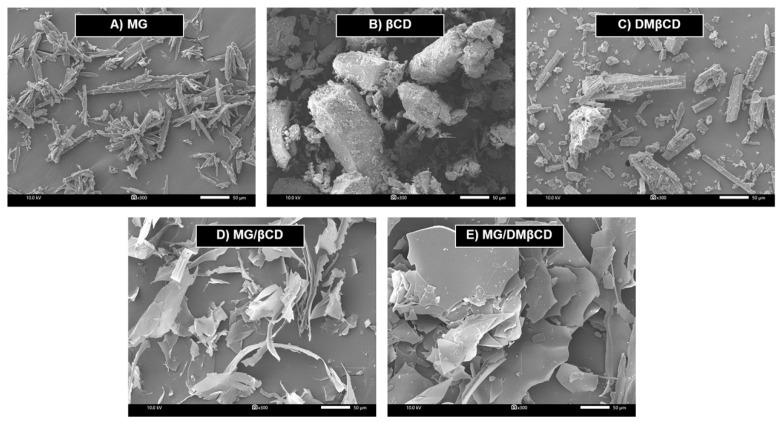
SEM images of (**A**) MG, (**B**) βCD, (**C**) DMβCD, (**D**) MG/βCD, and (**E**) MG/DMβCD at 300 times magnification.

It was evident that the inclusion complex formation dramatically induces the alterations of the particle shape and surface morphology of the resulting products [65,66,67]; thus, we performed SEM analysis in order to characterize the morphological changes upon host-guest encapsulation. The SEM micrographs at 300 times magnification of all investigated samples are summarized in Figure 7. In the case of the unbound forms, βCD and DMβCD are defined as rod-shaped structure, in accord with previous reports [68,69], whereas MG displays a flake-like feature. Upon molecular complexation, the surface morphology and the particle shape/size of the obtained freeze-dried inclusion complexes MG/βCD and MG/DMβCD (Figure 7D,E) are totally different from those of the free forms, confirming the successful formation between MG and βCDs. 

### 3.7. Cytotoxicity of MG/βCDs Inclusion Complexes toward Lung Cancer

**Figure 8:**
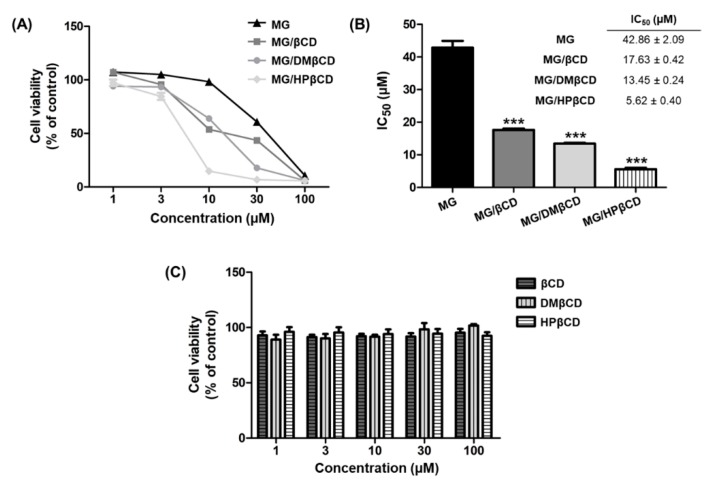
(**A**) Cell viability of A549 human lung cell line after treatment with logarithmic concentrations (1, 3, 10, 30, and 100 µM) of MG, MG/βCD, MG/DMβCD, and MG/HPβCD for 48 h. (**B**) The IC_50_ (µM) of all investigated compounds. (**C**) Cell viability of A549 cells treated with the free form of βCD, DMβCD, and HPβCD. Data are expressed as mean ± SEM of three independent experiments. *** *p* ≤ 0.001 vs. MG.

Lung cancer is the first leading cause of cancer-related death globally with its five-year survival rate of only 17.8% [70]. In this study, we evaluated the cytotoxic activity of MG and its inclusion complexes (MG/βCD, MG/DMβCD, and MG/HPβCD) against A549 human lung cancer cell line using MTT assay. The cell viability (% of control) results are summarized in Figure 8A, whereas the IC_50_ values (μM) are shown in Figure 8B. The obtained results reveal that MG, MG/βCD, MG/DMβCD, and MG/HPβCD decrease cell viability in a concentration-dependent manner, in which MG/HPβCD exhibits the lowest cell viability followed by MG/DMβCD, MG/βCD, and MG, respectively (Figure 8A). 

Multiple lines of evidence have shown that βCD inclusion complexations can potentially enhance the antitumor activity of lipophilic guest compounds [25,67,71]. In correlation with these reports, the present study shows that the inclusion complexes of MG significantly increase the anticancer effect on A549 cells, which can be ranked in the order of MG/HPβCD (IC_50_ of 5.62 ± 0.40 μM, ***) > MG/DMβCD (IC_50_ of 13.45 ± 0.24 μM, ***) > MG/βCD (17.63 ± 0.42 μM, ***) >> MG (42.86 ± 2.09 μM) (Figure 8B). The enhancement of anticancer activity of MG by complexation with βCDs is likely due to the reason that βCDs can infiltrate into the drug permeation barrier, called unstirred water layer (UWL) consisting of a large number of strong H-bond networks [72], better than the free form of lipophilic MG. It is likely that the introduced hydrophilic HP (HPβCD) and the hydrophobic methyl groups on βCD (DMβCD) could respectively (*i*) enhance the flux of drug though UWL [73] and (*ii*) interact with biological membrane greater than unmodified CD [74]. Therefore, the cytotoxic effect on A549 of MG/HPβCD and MG/DMβCD is higher than that of MG/βCD. Importantly, the free form of βCD, DMβCD, and HPβCD does not affect the antitumor property of MG/βCDs inclusion complexes, as evidenced by the cell viability values of >90% (Figure 8C). 

## 4. Conclusions

In the present study, we combined theoretical and experimental studies to identify the most suitable βCD host molecule for MG, a promising anticancer agent extracted from *Mansonia gagei*, for improving the aqueous solubility and the antitumor potential. The 90-ns MD simulations in triplicate revealed that MG preferentially positioned inside βCD, DMβCD, and 2HPβCD cavities through its aromatic ring. The MG binding led to the rigidity of βCDs by increasing the intramolecular H-bond formations on the wider rim. Among the three different MG/βCDs, the Δ*G*_bind_ values from MM/GBSA calculations and experimental phase solubility study revealed that MG/DMβCD was the most stable complex, as supported by the lowest water accessibility toward MG atomic radii and the highest number of contacts between host and guest molecules. The successful formation of MG/βCDs was confirmed by DSC and SEM techniques. The anticancer activity of MG toward A549 lung cancer cells was significantly enhanced (~2–7 fold) by complexation with βCDs, especially HPβCD and DMβCD analogs. Altogether, the obtained results confirmed the working hypothesis and demonstrated the good potentiality of βCD derivatives as suitable formulations of MG for further pharmaceutical and medicinal applications.

## Figures and Tables

**Table 1:** The average Δ*G*_bind, MM/GBSA_ and its energy components (kcal/mol) of MG/βCDs inclusion complexes. Data are expressed as mean ± SEM from three independent MD simulations. Δ*E*_MM_, molecular mechanics energy; Δ*G*_solv_, solvation free energy comprising polar (Δ*G*_solv,polar_) and non-polar (Δ*G*_solv,non-polar_) terms; Δ*S*, entropy. * *p* ≤ 0.05 and *** *p* ≤ 0.001 vs. MG/βCD.

Energy Component (kcal/mol)	MG/βCD	MG/DMβCD	MG/2HPβCD
Δ*E*_vdW_	−27.61 ± 1.19	−29.19 ± 0.29	−28.59 ± 0.64
Δ*E*_ele_	−11.79 ± 0.40	−4.43 ± 0.19	−6.40 ± 0.90
Δ*E*_MM_	−39.40 ± 1.58	−33.62 ± 0.26	−34.99 ± 0.26
Δ*G*_solv,polar_	22.14 ± 0.91	12.72 ± 0.13	17.17 ± 0.54
Δ*G*_solv,non-polar_	−2.83 ± 0.04	−2.98 ± 0.01	−3.10 ± 0.02
Δ*G*_solv_	19.31 ± 0.86	9.73 ± 0.12	14.06 ± 0.57
Δ*G*_solv,polar_ + Δ*E*_ele_	10.35 ± 0.53	8.29 ± 0.19	10.77 ± 0.47
Δ*G*_solv,non-polar_ + Δ*E*_vdW_	−30.44 ± 1.24	−32.18 ± 0.31	−31.69 ± 0.66
TΔ*S*	−17.74 ± 0.36	−18.16 ± 0.13	−17.57 ± 0.42
Δ*G*_bind, MM/GBSA_	−2.34 ± 0.35	−5.73 ± 0.04 (***)	−3.35 ± 0.14 (*)

**Table 2:** *Kc* of MG/βCDs inclusion complexes at different temperatures. Note that the 95% confidence interval and the other statistic values for the slope and the y-intercept of linear regression are shown in Table S2.

Temperature (°C)	Stability Constant (*Kc*, M^−1^)		
	MG/βCD	MG/DMβCD	MG/HPβCD
30	562	2245	684
37	165	643	245
45	109	358	173

**Table 3:** Thermodynamic values for the inclusion complex formation of MG with βCDs derived from Van’t Hoff plots (using R of 1.985 × 10^−3^ kcal·mol^−1^·K^−1^ and T of 303 K) in comparison to the Δ*G*_bind, MM/GBSA_. Note that the 95% confidence interval and the other statistic values for the slope and the y-intercept of linear regression are shown in Table S3.

Thermodynamic Parameter (kcal/mol)	MG/βCD	MG/DMβCD	MG/HPβCD
∆*H*	−20.77	−23.27	−17.39
T∆*S*	−17.06	−18.71	−13.54
∆*G*_bind, exp_ (30 °C)	−3.71	−4.56	−3.85
∆*G*_bind__, MM/GBSA_ (Table 1)	−2.34 ± 0.35	−5.73 ± 0.04	−3.35 ± 0.14

**Table 4:** Solubility of MG and its inclusion complexes at 30 °C.

	Solubility of MG (mg/L)
MG	1.7
MG/βCD	11.5
MG/DMβCD	47.2
MG/HPβCD	17.1

## Supplementary Materials

The following are available online at https://www.mdpi.com/2218-273X/9/10/545/s1, Figure S1: Van’t Hoff plots of MG/βCDs inclusion complexes., Figure S2: DSC thermogram of MG, βCD, DMβCD, and the physical mixtures MG/βCD and MG/DMβCD. Note that prior to perform DSC, all compounds were not dissolved in deionized water and were not subsequently lyophilized., Table S1: Linear equation of MG/βCDs inclusion complexes at different temperatures derived from Figure 5., Table S2: The 95% confidence interval and the other statistic values for the slope and the y-intercept of linear regression of phase solubility study derived from Figure 5., Table S3: The 95% confidence interval and the other statistic values for the slope and the y-intercept of linear regression of Van’t Hoff plot derived from Figure S1.
