# Supplementary material for: Enhanced Solubility and Anticancer Potential of Mansonone G By β-Cyclodextrin-Based Host-Guest Complexation: A Computational and Experimental Study
Source: Biomolecules. 2019 Sep 27;9(10):545. doi: 10.3390/biom9100545 (PMC6843486; doi:10.3390/biom9100545)
Supplement: Supplementary file 1 [file biomolecules-09-00545-s001.pdf]

Supplementary data

Table S1. Linear equation of MG/βCDs inclusion complexes at different temperatures derived from **Figure 5**.

Temperature (ºC)	Y= mX + c			r^2		
	**MG/βCD**	**MG/DMβCD**	**MG/HPβCD**	**MG/βCD**	**MG/DMβCD**	**MG/HPβCD**
**30**	Y=0.0040X+0.0072	Y=0.0180X+0.0082	Y=0.0063X+0.0093	0.996	0.993	0.993
**37**	Y=0.0041X+0.0249	Y=0.0164X+0.0259	Y=0.0065X+0.0268	0.995	0.995	0.999
**45**	Y=0.0050X+0.0459	Y=0.0189X+0.0537	Y=0.0088X+0.0509	0.997	0.988	0.981

Table S2. The 95% confidence interval and the other statistic values for the slope and the y-intercept of linear regression of phase solubility study derived from **Figure 5**.

		Standard Error	*t Stat*	*P-value*	*Lower 95%*	*Upper 95%*
**MG/βCD**						
**30ºC**	**Intercept**	0.0006	10.99	0.0003	0.0054	0.0090
	**Slope**	0.0001	37.24	0.0000	0.0037	0.0043
**37ºC**	**Intercept**	0.0008	31.36	0.0000	0.0227	0.0271
	**Slope**	0.0001	31.14	0.0000	0.0037	0.0044
**45ºC**	**Intercept**	0.0008	60.02	0.0000	0.0438	0.0480
	**Slope**	0.0001	39.33	0.0000	0.0046	0.0053
**MG/DMβCD**						
**30ºC**	**Intercept**	0.0041	2.008	0.1151	-0.0031	0.0194
	**Slope**	0.0007	26.80	0.0000	0.0161	0.0198
**37ºC**	**Intercept**	0.0031	8.415	0.0011	0.0174	0.0345
	**Slope**	0.0005	32.22	0.0000	0.0150	0.0178
**45ºC**	**Intercept**	0.0057	9.510	0.0007	0.0380	0.0694
	**Slope**	0.0009	20.23	0.0000	0.0163	0.0215
**MG/HPβCD**						
**30ºC**	**Intercept**	0.0014	6.466	0.0029	0.0053	0.0132
	**Slope**	0.0002	26.61	0.0000	0.0056	0.0069
**37ºC**	**Intercept**	0.0006	44.06	0.0000	0.0251	0.0285
	**Slope**	0.0001	64.92	0.0000	0.0062	0.0068
**45ºC**	**Intercept**	0.0033	15.32	0.0001	0.0418	0.0602
	**Slope**	0.0005	15.92	0.0000	0.0072	0.0103

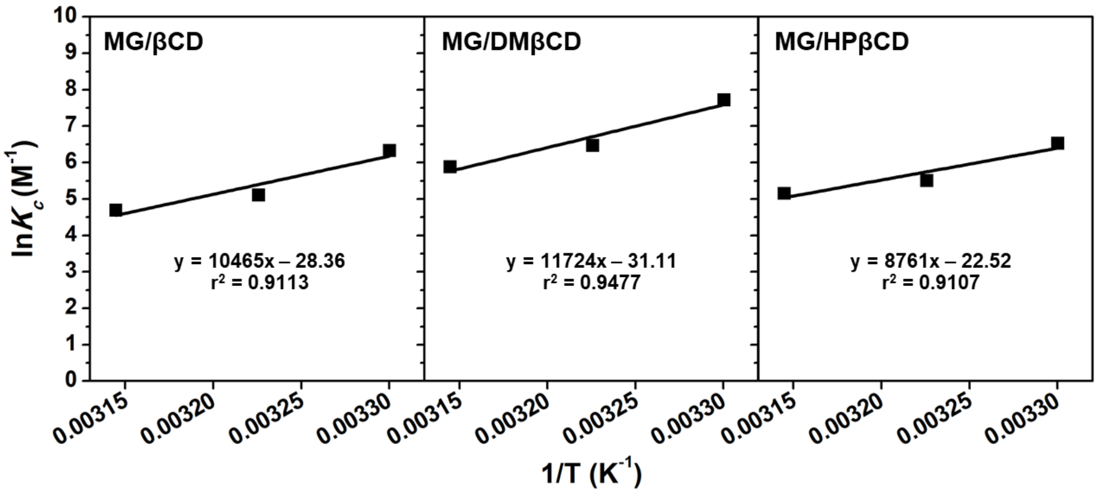

Figure S1. Van't Hoff plots of MG/βCDs inclusion complexes.

Table S3. The 95% confidence interval and the other statistic values for the slope and the y-intercept of linear regression of Van't Hoff plot derived from **Figure S1**.

	Standard Error	*t Stat*	*P-value*	*Lower 95%*	*Upper 95%*
**MG/βCD**					
**Intercept**	10.53	-2.695	0.226	-162.1	105.4
**Slope**	3265	3.206	0.193	-31016	51947
**MG/DMβCD**					
**Intercept**	8.877	-3.504	0.177	-143.9	81.69
**Slope**	2753	4.258	0.147	-23259	46709
**MG/HPβCD**					
**Intercept**	8.846	-2.545	0.238	-134.9	89.88
**Slope**	2743	3.194	0.193	-26098	43621

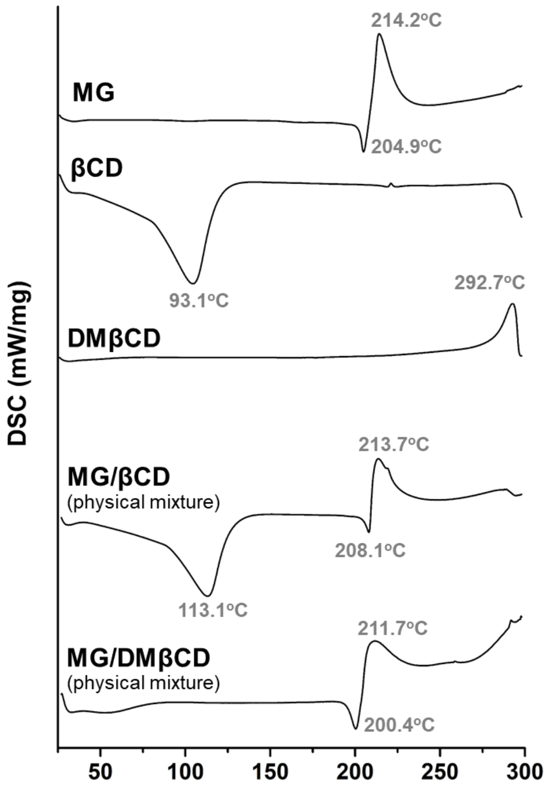

Figure S2. DSC thermogram of MG, βCD, DMβCD, and the physical mixtures MG/βCD and MG/DMβCD. Note that prior to perform DSC, all compounds were not dissolved in deionized water and were not subsequently lyophilized.
